# Pathology of Alveolar Abscess

**Published:** 1885-12

**Authors:** W. H. Whitslar


### THE DENTAL REGISTER.

VOL. XXXIX.] DECEMBER, 1885. [No. 12.

### COMMUNICATIONS.

### Pathology of Alveolar Abscess.

BY W. H. WHITSLAR, D.D.S.

Read before the Northern Ohio Dental Association.

In no bone or group of bones, arranged for the support or protection of any organ or set of organs in the human body, do we find so vast an array of alveoli as in the maxillary bones of the face. Averaging forty-eight in number, sometimes more and sometimes less, including the alveoli of both jaws, it is from this great number that we have been led to speak of any disease limited by the word alveolar, as that pertaining to any one of the alveoli contained in the alveolar processes of either maxilla. Hence when there occurs within an alveolus an abscess we usually name it alveolar from its position and not its kind.

In the study of the pathology or physiology of alveolar abscess, we learn much of its history when we study life in its simpler forms, and it is for this purpose that a short *resume* is made in a brief analysis. Therefore we take in view a simple cell, a mere mass of protoplasm, without definite form or organism, and what do we find? We discern, first, that man with his grand combination of bones, muscles, vascular and nervous system, does not exhibit in proportion to size as much energy as that of the ever changing bit of protoplasm; secondly, that the forces which are so apparent in higher life are in all probability the same in the lower. Chemical application cannot detect this, for, when an attempt is made to separate the component parts of so tiny a mass, microscopic in size, the elements which are in so minute quantities cannot be collected into separate vessels without severe disturbing influence interfering, which at once renders the separation impossible. And then too, protoplasm being so complex, all methods of chemical analysis destroy it, and what we analyze is *not* protoplasm, but a mixture of the products of its decomposition. Such a mixture has been called *dead* protoplasm, but that term implies a contradiction since protoplasm is *always* alive. Now man and a simple cell possess in common with inanimate objects many merely physical properties, as weight, color, and so on, but, there are at least seven well known features that are recognizable in the life of man in the highest sphere of the animal kingdom and the amœba unknown without the aided eye. These features or qualities are, irritability, contractibity, conductivity, co-ordination, automatism, assimilation, and reproduction; and at times are especially endowed more than another in certain directions. Of the first, irritability is noticed when foreign bodies come in close contact, which results in movements of the mass from the co-ordination of contraction and conductivity. With contraction there seems to be an identity with the muscle movements of our own bodies, that is, the fundamental principles seem to be the same. Conductivity necessarily follows irritability since impingement upon a part not only produces local effects but distant parts co-operate. This is appreciated through our nervous systems. Automatism is action set up in itself, and is dependent strictly upon nutritional powers. The remaining two factors, assimilation and reproduction, are possessed by all kinds of living matter, whether plant or animal. There is, however, an important distinction between the two, and that is, while assimilation is necessary for the maintenance of life, reproduction on the other hand is necessary only for the continuance of a race or kind, and if need be, is possessed only by some of the individuals composing it.

All these virtues then are recognizable in the simple cell as well as in man, and why not? Dissection of our bodies into their histological elements shows that man is but a federation of cells, and that all are arranged with some great end in view; cells of one kind to perform certain functions, cells of another to accomplish other designs. Every human body commences its individual existence as a single nucleated cell. These cells are soft and granular, with a central portion known as the nucleus, and, a still smaller spot is noticed within the nucleus and is called the nucleolus. Professor Agassiz gives to the granular matter the name of *ectoblast*, to the nucleus *mezoblast*, and to the nucleolus *entoblast*. The nucleus is the living part of the cell, and has within it the power of division or reproduction. The outer part is the dead matter which when removed from its source of nutrition is cast off after a certain age. If an epithelial surface be examined, selecting that which is near a blood capilliary, we notice that those cells which are nearest the capillary are composed of nucleus matter only, these of course are germinal, and all the future parts of the cell must accrue from them no matter how complex. Examine a little further away and as seen in the diagram the cells have appearing upon their outer edges, a new material forming still further away, this increases in quantity while at the same time the nucleus becomes smaller and smaller until there ceases to exist at all any of the living matter. Growth stops when the weight of egesta equals the amount of ingesta. Finally the entire cell is composed of formed material or effete matter. So much for living matter, its growth, and death; but what is true of one member of a congregation of cells in life is equally true of the entire number under the same conditions. Hence when we deal with a tooth and its surrounding tissues the same laws govern their retention or expulsion as nature seems fit to choose. Now, coming more directly to the subject in hand, we recognize that an abscess is the result, (if not controverted,) of inflammation. Inflammation implies much, and its study is one of the most difficult that the surgeon can enter upon.

Burdon Sanderson in 1870, gave a definition of inflammation which is now most universally accepted. It is this: "Inflammation is the succession of changes which occurs in living tissue when it is injured, provided that the injury is not of such a degree as at once to destroy its structure and vitality." With this definition in view then, we will conceive a case of alveolar abscess located at or near the apex of the root of a tooth. This abscess is, as we shall see, one of the succession of changes occuring from a violent inflammatory process taking place in the alveolo dental periosteum. During the process of the formation of this abscess we perceive three distinctive changes; 1st, in the blood-vessels and blood, 2nd, in the tissues, 3d, in the nerves.

An injury to the part having been established, nature ever ready to repair the damage done by any cause, sends nutriment to the part and at once gives up its energy to resist the attack. It is through the blood that we find this avenue of repair. Hence what we first notice is a sudden contraction of the vessels in the vicinage, produced by a reflex action through the vaso-motor centre; then inhibition follows or perhaps a mere relaxation, and then comes an increased flow of blood to the part. There is naturally a slight exudation of blood plasma from the vessels, but osmosis of this fluid becomes greater when the vessels become hyperæmic. Blood-pressure is the great law governing osmosis of fluids from the blood-vessels, and when the vessels become over full, a greater amount of the liquor sanguinis transudes. With this transudation there is also a migration of the white blood corpuscles, and this is a peculiar process. We have in the flow of blood through the vessels what may be said to be two currents; that which moves faster in the middle of the stream or *axial* current, and that at the sides which, from the great resistance of its bed and walls moves slower and is called the *inert* current. Now, white corpuscles seem to remain in the inert current, and cling to the walls of the vessels (in disease.) This is a physical phenomenon due to the irregular form of the corpuscles themselves, and the power they have of making amœboid movements. The red corpuscles choose the axial current. When from the rush of blood the vessels become clogged, the white corpuscles are seen to pass through their walls. At first there is noticed a slight elevation upon the outer part of the vessel opposite the corpuscle, then it enlarges and becomes a hemispherical prominence corresponding to the width of the remainder of the corpuscle within the vessel. As the external part increases, that on the inside diminishes, and finally the entire corpuscle bursts through on the outer side. The peculiarity of this process is that the vessels do not lose their contour and no rupture of their coats is visible.

As a result of this process the tissues become infiltrated with the leucocytes which assume different shapes and forms. It is only in a very violent stage of the disease that the red corpuscles pass through the coats of the vessels. Besides all these changes the vessels themselves are weakened and lack tonicity.

The changes in the tissues : First we note the condition of the membranes surrounding the tooth, and the first aspect we look at should be viewed in its anatomy. Many mistake the true significance of this membrane and seem to insist that it is a periosteum reflected from the alveolus upwards upon the sides of the tooth. To review the subject, I beg leave to invite your attention to a diagramatic drawing showing the direction of the fibres of connective tissue forming the membrane from the walls of the alveolus to the cementum of the tooth. You will notice that they do not pass directly transverse but obliquely across. With inflammatory disease these fibres become swollen, and coupled with the swollen condition of the blood-vessels it is readily understood how a tooth is raised from its socket. Such a feeling may however be experienced from a hyperœsthia of these nerves.

Tomes says that the membrane is quite cellular near the cementum. If such be the case how easily it is that plastic exudation and blood corpuscles will pass into its meshes, as shown by the diagram, represents cellular tissue inflated and completely infiltrating that tissue. Such is the case exactly just previous to the formation of pus, an essential element of an abscess. For it is by the coagulation of this plastic exudation or lymph, that a clot is formed incorporating in it the blood corpuscles and making a pocket for the retention of pus. This clot we know as inflammatory lymph. A collection of pus in such a cavity is a *true* abscess, and extension takes place by a progressive destruction of the tissues. Pus of itself is formed by successive zones of new cells degenerating, becoming separated by fluids and falling into the cavity of the abscess. This zone of tissue in which the process takes place was formerly called "pyogenic membrane," but the latest writer upon the subject calls it "pyogenic zone," which is a more correct term.

Pus, as you know, presents a considerable variety in character according to the cause that gives rise to its formation. Healthy or laudable pus contains about ninety per cent. of water. The solid constituents consist of two-thirds albumen and the remaining third fats, and some salts formed usually in blood serum. Then we have the ichorous or thin and watery, the sanious or bloody, and if diluted with mucus the muco-pus. Microscopically pus presents a clear watery fluid with numberless corpuscles, many resembling the white blood corpuscle, of which only a very few are living.

In particular we notice two forms of abscess about a tooth, and these are the acute and chronic. The acute is taken as the type. In this the pain is of a throbbing nature; the parts become œdematous with some fluctuation; the gums become very red and shiny with a still darker ring of red about the neck of the tooth. In the chronic form swelling is the prevailing symptom with some dull pain. In both cases the collection of pus, swelling of the tissues, and the surplus liquid transudation, together produce the third change as a result of abscess, and this is the effect upon the nerves. This we might say is a *pressure* effect. The sequence of it is pain, either immediate or spasmodic in remote parts.

Thus we have endeavored to bring within the narrow limits of this paper a *few* points bearing upon the physiology of alveolar abscess, knowing full well that such a subject demands a greater amount of research and knowledge than I am able to give.

